# Long chain polyunsaturated fatty acids (LCPUFAs) and nordihydroguaiaretic acid (NDGA) modulate metabolic and inflammatory markers in a spontaneous type 2 diabetes mellitus model (Stillman Salgado rats)

**DOI:** 10.1186/s12944-016-0363-8

**Published:** 2016-11-25

**Authors:** Alejandro Dain, Gaston Repossi, Gustavo T. Diaz-Gerevini, Jairam Vanamala, Undurti N. Das, Aldo R. Eynard

**Affiliations:** 1 Biología Celular, Histología y Embriología, Facultad de Ciencias Medicas, INICSA (CONICET-Universidad Nacional de Córdoba), Córdoba, Argentina; 2 Cátedra de Histología, Embriología y Genética, Universidad Nacional de La Rioja, La Rioja, Argentina; 3 CONICET, Córdoba, Argentina; 4 Department of Food Science, Penn State University, 326 Food Science Building, University Park, PA 16802 USA; 5 UND Life Sciences, 2020 S 360th St, # K-202, Federal Way, WA 98003 USA; 6 BioScience Research Centre and Department of Medicine, GVP Hospital, Gayatri Vidya Parishad College of Engineering Campus, Visakhapatnam, 530 048 India

**Keywords:** Type 2 diabetes, eSS rats (Stillman Salgado rats), PUFAs, Chronic inflammation, Oxidation process, Plasma triglycerides, Nordihydroguaiaretic acid

## Abstract

**Background:**

Diabetes mellitus (DM) is a complex disease with alterations in metabolic and inflammatory markers. Stillman Salgado rats (eSS) spontaneously develop type 2 DM by middle age showing progressive impairment of glucose tolerance with hyperglycemia, hypertriglyceridemia and hyperinsulinemia. We analyzed the effects of supplementation of ω-3 and ω-6 polyunsaturated fatty acids (PUFAs) with or without nordihydroguaiaretic acid (NDGA) added, an antioxidant and lipoxygenase inhibitor, on metabolic and inflammatory parameters in eSS rats to evaluate whether they can delay development and/or prevent progression of DM.

**Methods:**

After weaning, eSS rats received, intraperitoneally, once a month ω-3 (EPA 35% and DHA 40%–6.25 mg/Kg) or ω-6 (90% arachidonic acid- 6. 25 mg/Kg) for twelve months. Two additional groups of rats received 1.9 mg/kg NDGA added to ω-3 and ω-6 fatty acids. Blood samples were collected at day 40, and at the end of the 6th month and 12th month of age to determine plasma triglycerides (TGs), total plasma fatty acids (FA), A1C hemoglobin (HbA1C), C-reactive protein (CRP), gamma glutamyl transpeptidase (GGT), lipo and hydro peroxides, nitrites and IL-6 (in plasma and liver, kidney, and pancreas) and underwent oral glucose tolerance test (OGTT) as well. Wistar and eSS rats that received saline solution were used as controls.

**Results:**

Plasma lipids profile, TG, fasting and post-prandial blood glucose levels, and glycosylated HbA1C showed significant improvements in ω-3 and ω-3 + NDGA treated animals compared to eSS control group. ω-3 and ω-3 + NDGA groups showed an inverse correlation with fasting blood glucose and showed lower plasma levels of GGT, TG, and CRP. eSS rats treated with ω-3 LCPUFAs showed reduced level of inflammatory and oxidative indices in plasma and liver, kidney and pancreas tissues in comparison with eSS control (non-treated) and ω-6 treated groups.

**Conclusions:**

eSS rats are a useful model to study type 2 DM pathophysiology and related inflammatory indices. ω-3 + NDGA supplementation, at the doses tested, ameliorated inflammatory, metabolic and oxidative stress markers studied.

## Background

DM is a complex disease in which alterations in metabolic and inflammatory indices including perturbations in the metabolism of glucose, lipids and proteins occur. Perturbations in the oxidative cycle and cellular stress and alterations in glucose metabolism result in an elevation of inflammatory markers: interleukins-2 and 6 (IL-2 and IL-6), leukotrienes (LTs such as LTB4), and C-reactive protein (CRP) [1]. The increasing incidence of DM not only impacts the health of the affected individual but also enhances the cost of health care and has implications for political, economic, and social issues of the society [2]. DM is estimated to affect about 366 million by 2030. DM and obesity have common pathophysiological pathways that may occur due to inadequate physical activity and consumption of high-calorie/high-fat food intake that results in insulin resistance and metabolic syndrome [3]. It has been reported that an imbalance in the metabolism of ω-3 and ω-6 long-chain polyunsaturated fatty acids (LCPUFAs) occurs in obesity, insulin resistance, metabolic syndrome, and DM [4, 5].

eSS rats are a strain derived from inbred Wistar rats, which develop spontaneously type 2 DM without obesity that resembles closely type 2 DM seen in adult humans. Type 2 DM is more severe in male eSS rats and they survive an average of 18 months if insulin is not administered to control hyperglycemia. In early stages of the development of DM, eSS rats show glucose intolerance with hyperinsulinemia and dyslipidemia. These findings are similar to those observed in humans with type 2 DM [6–8].

In the present study, we administrated ω-3 (fish oil rich in EPA 35% and DHA 40% obtained from Natufarma® Argentina) and ω-6 (AA 90% Sigma®) PUFAs with and without nordihydroguaiaretic acid (NDGA), and studied their effects on metabolic and inflammatory indices. NDGA is a natural product extracted and isolated from native shrub specie of *Larrea sp.* NDGA inhibits predominantly lipoxygenase (LOX) and partially, cyclooxygenase (COX) pathways with powerful anti-inflammatory, anti-apoptotic and anti-oxidative actions [9, 10]. It is believed that inhibition of LOX and COX pathways and administration of anti-inflammatory compounds may be of benefit in type 2 DM especially in preventing long-term complications of DM especially those related to inflammatory and oxidative stress related complications that are generally mediated by IL-6, tumor necrosis factor-α (TNF-α), prostaglandin E2 (PGE2, derived from arachidonic acid), reactive oxygen species (ROS) and other related molecules. It has been postulated that ω-3 PUFAs are capable of suppressing IL-6, TNF-α, PGE2, and ROS production and thus, may be of benefit in type 2 DM. Hence, we studied the effect of ω-3 PUFAs with and without NDGA on various inflammatory and oxidative stress indices in eSS rats. We have chosen intraperitoneal route to administer PUFAs and NDGA because it allows to deliver the exact amount of the desired substance without loss or unintentional spills and to bypass possible influences of gut enzymes, gut microbiota and dietary fiber among others on the chosen chemicals that are employed to study [5, 11–16].

The results of this study showed that intraperitoneal administration of ω-3 LCPUFAs and, especially that of a combination of ω-3 + NDGA decreased oxidative and inflammatory markers and improved metabolic parameters in this eSS model of spontaneous type 2 DM.

## Results and discussion

### Weight

**Fig. 1:**
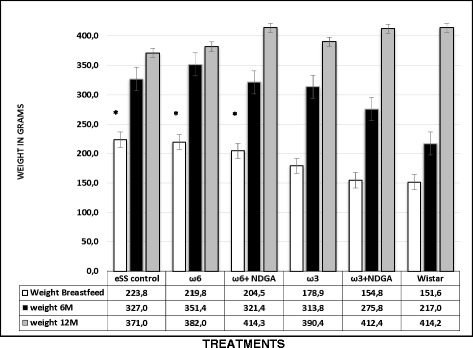
Weight changes (in grams) in eSS male rats at the end of breast feeding, 6 months and 12 months of age. *Indicate significant difference of Wistar at breastfeed p<0.05

It was observed that breast-fed eSS rats had a higher body weight compared to Wistar rats till the age of 6 months. But, this difference in their body weights disappeared at 6^th^ and 12^th^ months (Fig. 1).

#### Plasma lipid profile

**Fig. 2:**
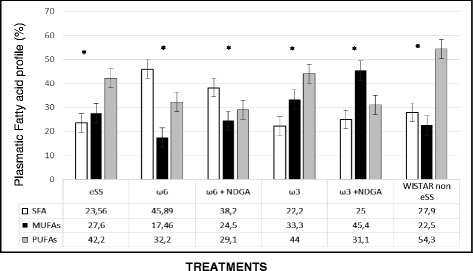
Plasmatic total fatty acids profile by GLC in experimental groups of rats at 12th month of age, SFA (saturated fatty acids), MUFAs (mono unsaturated fatty acids) and PUFAs (Poli unsaturated fatty acids)

**Fig. 3:**
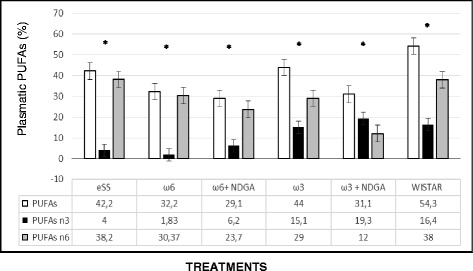
Plasmatic PUFAs ω 3 and ω 6 levels by GLC at 12th month of age in experimental groups of rats

Quantitative and qualitative differences in the lipid profile of experimental groups are shown in Figs. 2 and 3, and in Tables 1 and 2. The eSS rats showed significant alterations in their lipid profile as has been described previously [10, 11]. Clinical, experimental and epidemiological evidences established that lipid metabolism abnormalities are associated with diseases such as coronary artery disease, cancer and diabetes mellitus [17]. Our results showed total saturated fatty acids (SFA) values are significantly higher in the ω-6 group compared to the ω-3 group. Total monounsaturated FAs (MUFAs) were significantly higher in the ω-3 group in comparison to eSS control and ω-6 groups. Total ω-3 LCPUFAs were significantly higher in the ω-3+NDGA group, whereas gamma-linolenic acid (GLA 18:3n6) was significantly lower in the ω-3 group compared to eSS control and ω-6 groups.

**Table 1:** Fatty acid composition in the plasma at the end of 12^th^ month of age in eSS, Wistar and ω6 and ω3 LCPUFA supplemented eSS rats (%)

*FATTY ACID*	*eSS control*	*SDM*	*ω6*	*SDM*	*ω6 + NDGA*	*SDM*	*ω3*	*SDM*	*ω3 + NDGA*	*SDM*	*WISTAR non eSS*	*SDM*
14:0	0,5	0,2	1,16	0,5	0,9	0,6	0,6	0,05	0,7	0,8	1,6	1,3
16:0	14,49	4,7	32,26	10,6	26,5	11,7	15,4	0,2	18	8,4	13,4	6,4
18:0	8,14	3,9	12,17	2,7	10,6	0,4	6	1,1	6,5	2,4	10,9	2,1
24:0	0,43	0,3	0,3	0,02	0,2	0,007	0,2	0,02	0	0	1,9	0,3
*SFA*	23,56*	***9,1***	45,89* ^***#***^	***13,82***	38,2* ^***#***^	***12,7***	22,2* ^***#***^	***1,37***	25* ^***#***^	***11,6***	***27,9*** ^***#***^	***10,1***
14:1 n9	0,34	0,2	0,58	0,04	1,1	0,3	0,3	0,05	1,3	1,1	1,5	0,01
16:1 n7	5,18	4,1	1,66	0,9	6,8	1,5	9,8	0,3	31	1,6	1,3	0,2
18:1 n9	21,5	8,2	14,63	6,6	13,3	4,31	22,3	0,8	13	1,1	12,1	6,1
20:1 n9	0,31	0,2	0,34	0,007	2,2	0,3	0,8	0,1	0	0	5,3	0,4
22:1 n9	0,3	0,3	0,25	0,006	1	0,02	0	0	0	0	2,3	0,1
*MUFAs*	27,6*	***13***	17,46* ^***#***^	***7,553***	24,5* ^***#***^	***6,43***	***33,3*** ^***#***^	***1,25***	45,4*	***3,8***	***22,5*** ^***#***^	***6,81***
18:2 n6	23,7	5,8	19,75	0,08	15,2	2,7	27,6	0,1	8,6	1,4	17	9
18:3 n6	5	5,4	1,22	0,002	1,2	0,6	0	0	1,6	0,05	1,4	0,02
18:3 n3	1,5	0,6	0,22	0,001	1,3	0,3	3,9	0,5	13,6	1,5	1,5	0,9
20:2 n6	0,2	0,2	0,2	0.005	1,2	0,06	0,2	0,02	0	0	3,3	0,3
20:3 n3	1,2	0,7	0,08	0,01	1,5	1	8	0,2	2,3	0,5	3,7	0,2
20:4 n6	9,2	3,2	9,2	0,3	6,1	1	0,8	0,02	1,7	0,3	16,2	3,4
20:4 n3	0,1	0,1	0,2	0,01	0	0	2,3	0,05	0	0	3,7	0,2
20:5 n3	0,28	1,02	0,16	0,07	1,25	0,16	0,91	0,7	0,57	0,01	2,04	0,7
22:6 n3	0.0	0	0,2	0,01	1,1	0,3	0,2	0,01	0,2	0,02	5	0,4
22:5 n3	1,1	1,3	1,13	0,01	2,3	0,03	1,4	0,05	3,1	0,4	2,5	0,1
*PUFAs*	***42,2***	***18,3***	32,2*	***0,493***	29,1*	***6,15***	***44***	***1,65***	31,1*	***4,18***	***54,3***	***15,22***
*PUFAs n3*	4*	***2,9***	1,83*	***0,036***	6,2*	***1,63***	***15,1#***	***0,81***	19,3*#	***2,42***	***16,4*** ^***#***^	***1,8***
*PUFAs n6*	***38,2***	***14,4***	30,37* ^***#***^	***0,39***	***23,7*** ^***#***^	***4,36***	***29*** ^***#***^	***0,14***	***12*** ^***#***^	***1,75***	***38*** ^***#***^	***12,72***

Values represent means and Standard deviation of the mean (SDM). Values *P*< 0.05 were considered statistically significant. *Indicate significant difference of Wistar at 12th month. #Indicate significant difference of eSS control at 12th month

**Table 2:** Results of coefficient variance of total plasma fatty acids profile of experimental groups (Multivariate descriptive test T)

PARAMETER	eSS control	ω6	ω6 + NDGA	ω3	ω3 + NDGA	Wistar Control
p	0.0095	0.0229	0.0068	0.0112	0.0009	0.0003
COEF OF VARIANCE	145.14%	169.61%	153.45%	149.19%	147.34%	93.85%
SE	1.8	2.1	1.9	1.9	1.8	1.3

eSS control: Stillman Salgado rats without treatment; Wistar: non eSS rats without treatment. ω Groups: different PUFAs ω-3 and ω -6 treatments with or without NDGA (nordihydroguaiaretic acid). A value of *p*<0.05 was considered as significant

It is seen from the results of the present study that linoleic acid (LA, 18:2 ω-6) levels are higher while those of 20:3 ω-3, AA (20:4 ω-6), EPA (20:5 ω-3) and DHA (22:6 ω-3) are lower in eSS rats compared to Wistar control. Thus, in general, healthy Wistar control rats had much higher levels of long chain PUFAs compared to diabetic eSS rats. These results are similar to those seen in patients with type 1 and type 2 DM who are known to have lower levels of AA, EPA and DHA and altered ratios ω-6/ω-3 [18–20] compared to healthy controls. The increase in LA and decrease in its product AA in eSS rats compared to Wistar control indicates that the activities of Δ^6^ and Δ^5^ desaturases is altered in the eSS rats. It is surprising to note that plasma AA levels did not increase in eSS rats treated with ω-6, whereas it (AA) was decreased in ω-3 and ω-3 + NDGA groups.

These results could be explained, at least in part, by defective metabolic PUFAs pathway. Linoleic acid (LA, 18:2 ω-6) and α-linolenic acid (ALA, 18:3 ω-3), cannot be synthesized by mammals. Once incorporated by the cells, they can be desaturated and elongated to produce long chain PUFAs of the same family. Under normal conditions, ALA is preferentially desaturated and elongated to form eicosapentaenoic acid (EPA, 20:5 ω-3) and docosahexaenoic acid (DHA, 22:6 ω-3), whereas LA is also similarly desaturated and elongated to form γ-linolenic acid (GLA, 18:3 ω-3), dihomo-GLA (DGLA, 20:3 ω-6) and arachidonic acid (AA, 20:4 ω-6). It is known that both LA and ALA belonging to different families of PUFAs compete for the same set of enzymes of desaturases and elongases [18–20]. Changes in the activities of Δ^6^ and Δ^5^ desaturases in DM has been correlated to the lower content of AA and higher content of LA in almost all the tissues except brain [21, 22]. In experimental animals induced to develop type 2 DM and patients with type 2 DM, the changes in the activities of Δ^6^ and Δ^5^ desaturases have been variable [22–25], but data indicate that type 2 DM (at least in patients with long standing disease and poor glycemic control) negatively affects Δ^5^ desaturases activity and ω6/ω3 PUFAs balance [18, 26].

Administration of LCPUFAs (AA, EPA and DHA) to eSS rats can overcome this blockade in the PUFAs metabolic pathways and restore the PUFAs profile to normal as seen in healthy Wistar rats. Our results of the present study suggest that the enhanced levels of LA seen in eSS rats could be restored to levels seen in healthy Wistar rats by treating with AA (ω-6 and ω-6 + NDGA groups of the present study), and is in accordance with the previous results [19, 27]. The LCPUFAs derived from LA and ALA serve as precursors to several biologically active molecules such as prostaglandins (PGs), leukotrienes (LTs), thromboxanes (TXs), lipoxins (LXs), resolvins, protectins and endocannabinoids. These metabolites have potent pro-inflammatory or anti-inflammatory actions [28, 29]. The relative proportions of LCPUFAs in cell membranes, as well as cell type, are the primary factors that regulate the formation of some of these bioactive lipid metabolites. It is likely that eSS rats treated with ω-3 LCPUFAs may lead to the formation of some of these anti-inflammatory metabolites such as lipoxins, resolvins and protectins [28, 29]. This assumption is supported by our recent studies that showed that lipoxin A4, an anti-inflammatory product formed from AA has anti-diabetic actions in chemical-induced diabetic animal models [27].

In the present study, supplementation of ω-3 and ω-6 LCPUFAs to eSS diabetic rats did not reach levels of total PUFAs as detected in the plasma of healthy Wistar rats (Fig. 2 and 3). Despite this, evaluation of inflammatory and oxidative stress markers showed significant decrease in their concentrations.

#### Metabolic parameters

**Fig. 4:**
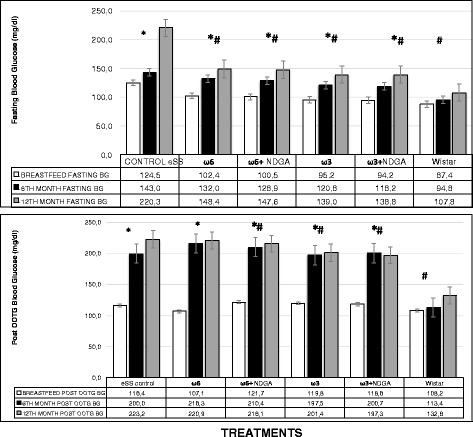
Fasting and Post OGTT blood glucose levels at the end of breast feeding, 6th and 12th months of age in experimental groups of animals, *Indicate significant difference of Wistar at 12th month p<0.05, ^#^Indicate significant difference of eSS control at 12th month p<0.05

**Fig. 5:**
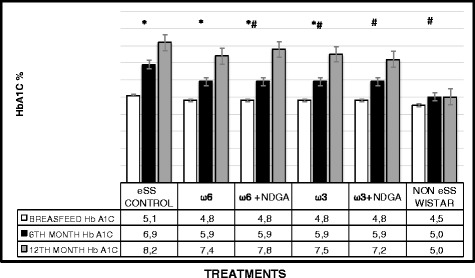
Glycosylated hemoglobin (HbA1c) levels at the end of breast feeding period, 6th and 12th month of age in experimental groups of animal, *Indicate significant difference of Wistar at 12th month p<0.05, ^#^Indicate significant difference of eSS control at 12th month p<0.05

**Fig. 6:**
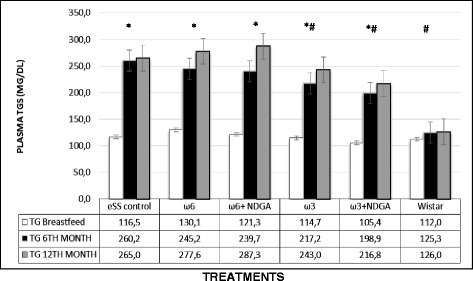
Plasma triglycerides levels at the end of breastfeeding, 6th and 12th month of age in experimental groups of rats, p<0.05, *Indicate significant difference of Wistar at 12th month, ^#^Indicate significant difference of eSS control at 12th month

Both fasting blood glucose (FBG) and post-prandial values in all the eSS groups were higher compared to the control Wistar group at the end of 6 and 12 months of age. Oral glucose tolerance test (OGTT) revealed that ω-3 and ω-3 + NDGA groups showed lower glycemic levels compared to other groups. In addition, HbA1C values were also found to be lower in ω-6 LCPUFA with or without NDGA groups compared to the untreated eSS control (Figs. 4 and 5). Addition of NDGA to ω-6 group also showed lower blood glucose levels in the OGTT test (Fig. 4). Blood TG levels were normal (ranged <150 mg/dl) in Wistar group, but its levels were higher at the end of 6 months onwards in all the eSS groups. eSS rats that received ω-6 LCPUFA (±NDGA) and eSS control groups showed higher TG levels, whereas ω-3 groups (±NDGA) had much lower levels (see Fig. 6), this fact is similar to that observed in patients with type 2 DM [11]. Plasmatic values of cholesterol were similar between the experimental groups and remained within normal values (data not show).

eSS rats develop type 2 DM at 6 months of age with a significant increase of A1C (31% above reference values) at the end of 12 months of age [19]. Glycosylated Hb (A1C) was measured on pre OGTT blood sample tests. Chronic elevation of HbA1c, an indicator that persistent hyperglycemia is present, has been strongly linked to higher mortality and poor prognosis in DM [30]. In the eSS control group, plasma TG levels were significantly increased prior to the development of DM. Higher plasma TG levels play a major role in lipotoxicity and modulate insulin sensitivity [31, 32]. Inflammatory and oxidative markers such as CRP and GGT were also significantly increased at 6 months of age in eSS rats even before an increase in fasting hyperglycemia occurred, suggesting that hyperlipidemia and pro-inflammatory events occur much before the development of clinical DM in the eSS model. This implies that insulin resistance seen in type 2 DM could be linked to alterations in oxidative stress and pro-inflammatory events. In ω-3 treated eSS group, plasma saturated FAs were significantly lowers as shown in Table 1. In addition, a significant decrease in fasting and post-prandial blood glucose was observed. HbA1c was significantly lower in both ω-6 and ω-3 LCPUFAs supplemented groups (23% in ω-3 groups and 26% in ω-6 groups, respectively) compared to Wistar controls, suggesting that the observed anti-inflammatory and antioxidant effects of ω-3 supplementation could result in an increase in insulin sensitivity as a result of enhanced expression of GPR120 (G-protein coupled receptor 120 is a protein that is encoded by the *GPR120* gene is a member of the rhodopsin family of G protein-coupled receptors). GPR120 mediates the anti-inflammatory and insulin-sensitizing effects of omega 3 fatty acids in the pancreatic beta cells and other target tissues such as liver, kidney, adipose tissue, and muscle. This could lead to increased glucose uptake, lipid storage and decreased circulating free FA [33, 34].

#### Inflammatory parameters

**Fig. 7:**
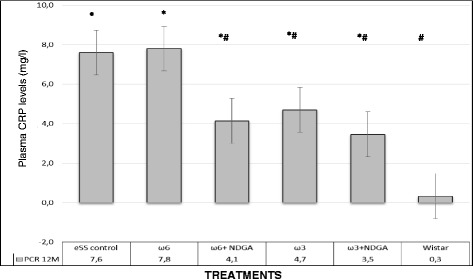
Plasmatic CRP levels in experimental groups of rats at 12th month of age, *Indicate significant difference of Wistar p<0.05, ^#^Indicate significant difference of eSS control at 12th month p<0.05

**Fig. 8:**
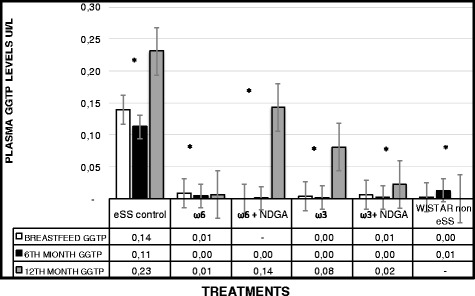
Plasma GGT levels at the end of breastfeeding, 6th and 12th month of age in experimental groups of rats

**Fig. 9:**
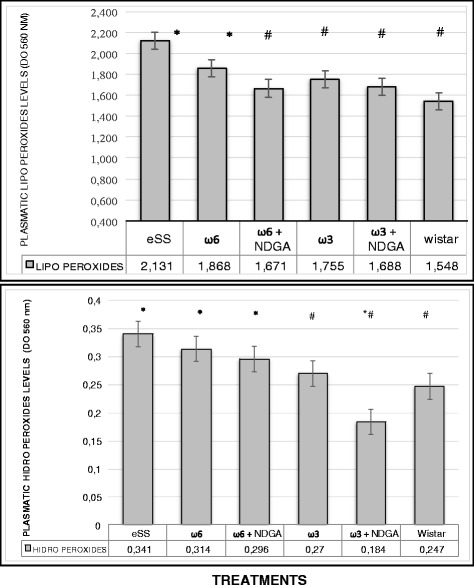
Plasmatic levels of Lipo and Hydro Peroxides at 12th month of age in experimental groups of rats, *Indicate significant difference of Wistar p<0.05, ^#^Indicate significant difference of eSS control at 12th month p<0.05

**Fig. 10:**
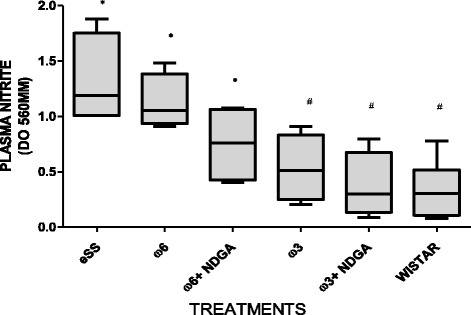
Plasma nitrite levels at the end of the12th month of age in experimental groups of rats, *Indicate significant difference of Wistar at 12th month, ^#^Indicate significant difference of eSS control at 12th month, p<0.05

**Fig. 11:**
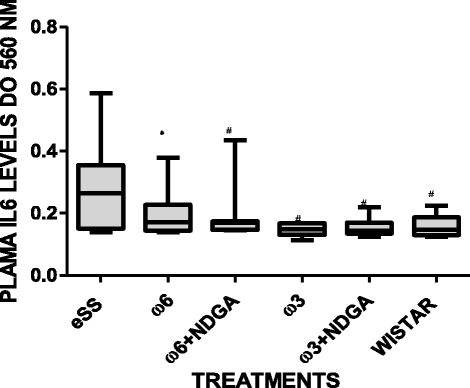
Plasma IL6 levels at the end of the12th month of age in experimental groups of rats, *Indicate significant difference of Wistar, ^#^Indicate significant difference of eSS control at 12th month, p<0.05

**Fig. 12:**
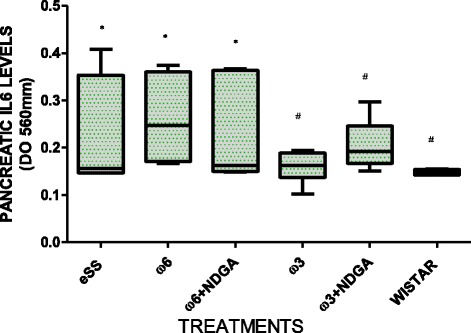
IL6 levels in pancreas of experimental rats at the end of the12th month of age, *Indicate significant difference of Wistar, ^#^Indicate significant difference of eSS control at 12th month, p<0.05

**Fig. 13:**
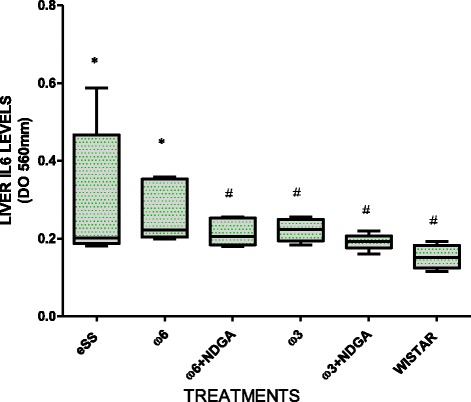
Liver IL6 levels at the end of the12th month of age in experimental groups of rats, *Indicate significant difference of Wistar, ^#^Indicate significant difference of eSS control at 12th month, p<0.05

**Fig. 14:**
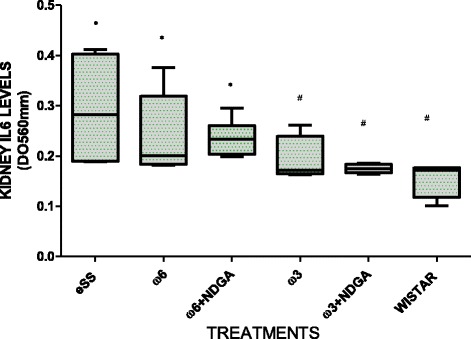
IL6 levels in kidney of experimental rats at 12th month of age, *Indicate significant difference of Wistar, ^#^Indicate significant difference of eSS control, p<0.05

Wistar rats showed normal plasma high sensivity C reactive protein (hs-CRP) values at the end of 12 months, while hs-CRP was significantly higher in the eSS control and ω-6 groups (Fig. 7). eSS control, ω-3 and ω-6 + NDGA groups showed increase in plasma GGT, however ω-3 + NDGA treatment resulted insignificant fall in their levels compared to the eSS control (Fig. 8). Similar decrease in the levels of IL-6, nitrites and peroxides was noted in ω-3 + NDGA group. In all groups that received ω-3 ± NDGA showed lower values with respect to peroxides (Fig. 9), nitrites (Fig. 10) and IL-6 (Figs. 11, 12, 13 and 14) compared to eSS rats and ω-6 groups. It is seen that plasma lipid and hydroperoxides, lipo peroxides, nitrites and IL-6 levels in groups that received ω-3 + NDGA were closer to those seen in Wistar control group (Figs. 9–14).

In ω-3 + NDGA treated eSS group, a synergistic action between these compounds reduced TG plasma values and NF-κB and JNK/AP1 expression by inhibition of TAK1 (Transforming growth factor β-activated kinase 1) that results in suppression of production of pro-inflammatory cytokines [31]. Other possible mechanisms of the beneficial action of ω-3 + NDGA could be due to its ability to act on PPAR-alpha and suppress expression of NF-kB [29]. On the other hand, it is also likely that ω-3 LCPUFAs act on GPR120 receptors as a consequence of which the expression of GLUT4 receptors is increased that leads to a decrease in hypertriglyceridemia and hyperglycemia by the suppression of inflammatory pathways [35, 36]. GPR120 has also been shown to mediate the anti-inflammatory and insulin-sensitizing effects of ω-3 LCPUFAs and its lack or defincency is responsible for reduced fat metabolism, thereby leading to obesity and DM [37]. It may also be noted here that ω-3 LCPUFAs may bring about their beneficial actions independent of GPR120 [38].

This is supported by the observation that the ω-3 treated eSS group with or without NDGA showed a significant reduction (>40%) in blood CRP at 12 months of age compared to the eSS control group. CRP is a sensitive pro-inflammatory marker, closely related to circulating IL-6, a cytokine that is released by activated macrophages, endothelial cells, adipocytes, muscle cells and T-lymphocytes to stimulate immune response [39]. IL-6 stimulates the inflammatory and auto-immune processes in many diseases such as diabetes, atherosclerosis, obesity, and cardiovascular diseases among others [40]. Results have shown a significant reduction in plasma and tissue (liver, kidney and pancreas) levels of IL-6 following ω-3 treatment (Figs. 11, 12, 13 and 14) and have reverted to near normal values found in the Wistar rats (reductions ≥50%). It is also noteworthy that eSS rats that developed insulin resistance and type 2 DM features showed evidence of systemic inflammation in the form of significantly elevated IL-6 levels not only in the plasma but also in various tissues examined (liver, pancreas and kidney) suggesting that insulin resistance, hyperlipidemia and type 2 DM are low-grade systemic inflammatory conditions as proposed previously [1, 5, 12, 41–43].

It has been suggested that an imbalance between ω-6/ω-3 LCPUFAs concentrations with a shift towards ω-6 LCPUFAs (ω-6 > ω-3) could contribute to the development of systemic low-grade chronic inflammation (SLGCI), which, in turn, may favor the initiation and perpetuation of endothelial dysfunction, insulin resistance, and consequently the development of hypertension and type 2 DM [44, 45]. PUFAs of ω-6/ω-3 families compete for the same set of enzymes and metabolic pathway, and are essential for the formation of long-chain metabolites like eicosanoids that have pivotal biological functions [5, 43]. AA, the major ω-6 LCPUFA which is known to be a precursor of predominantly pro-inflammatory eicosanoids (such as PGE2, PGF2α, TXA2 and leukotrienes) in significant amounts compared to the formation of less anti-inflammatory eicosanoids derived from ω-3 LCPUFAs, COX and LOX enzimes metabolize 20-carbons PUFA to produce eicosanoids and other bioactive lipids, its enzimes have more affinity by ω-3 PUFAs but ω-6 are usually in higher concentrations [5, 11]. Furthermore, PUFAs can also form precursors to anti-inflammatory compounds such as lipoxins, resolvins, and protectins. It is generally believed that under normal physiological conditions a balance is maintained between pro- and anti-inflammatory products formed to maintain normal homeostasis and suppress the initiation of low-grade systemic chronic inflammation in DM [5, 43]. In this context, it is noteworthy that ω-3 LCPUFAs induce their anti-inflammatory effects by acting on the GPR120 and Toll-like receptors (TLRs) [5, 28, 29, 36–38, 42, 43] that results in the suppression of formation of TNF-α and other pro-inflammatory cytokines especially in macrophages, adipocytes and hepatocytes [5, 28, 29, 43]. As a result, pro-inflammatory events are switched off or suppressed. In addition, NDGA is a potent antioxidant [9, 46, 47], and has potent anti-inflammatory actions by inhibition of COX-2 and LOX enzymes that results in decreased production of pro-inflammatory prostaglandins and leukotrienes. Hence, it is expected that a combination of ω-3 + NDGA may be more effective in suppressing inflammatory events as observed in the present study (see Figs. 7–14).

Oxidative markers such as plasma lipo- and hydro-peroxides and nitrites showed pronounced reductions when ω-3 + NDGA were administrated that were very close to the normal values seen in the non-diabetic Wistar rats. On the other hand, these values were closer to those seen in eSS control rats in the ω-6 treated groups. The ω-3 + NDGA treated eSS group showed decreased GGT at 6 and 12 months of age compared to ω-6 and eSS control groups. ω-3 and ω-6 LCPUFAs treated eSS groups, when co-supplemented with NDGA, showed much lower levels of GGT, CRP, IL-6, and peroxides at the time of fasting. This implies that NDGA supplementation has significant beneficial action in decreasing and attenuating inflammatory response and oxidative stress in this model.

#### Correlation study

Metabolic (TG), oxidative stress (GGT) and inflammatory parameters (CRP) were assayed at fasting and postprandial state after glucose administration that showed increased values in the eSS control whereas these indices were normal in Wistar control rats. ω-3 group (with or without NDGA addition) showed much lower values of GGT, TGs, CRP, IL-6 and lipid peroxides compared to ω-6 and eSS control groups (Table 3).

**Table 3:** Treatment correlation test (R2)

GROUP	PARAMETER	GGTP	TG	CRP	Plasma IL6	lipo Peroxides
eSS CONTROL	FASTING BLOOD GLUCOSE	0,93	0,66	0,45	0,88	0,24
	POSTPRANDIAL BLOOD GLUCOSE	0,25	0,89	0,81	0,94	0,54
ω6	FASTING BLOOD GLUCOSE	0,70	0,74	−0,70	0,32	0,65
	POSTPRANDIAL BLOOD GLUCOSE	−0,65	−0,13	−0,76	0,28	0,84
ω6 + NDGA	FASTING BLOOD GLUCOSE	−0,78	0,67	−0,14	−0,12	0,17
	POSTPRANDIAL BLOOD GLUCOSE	−0,51	0,84	−0,60	−0,08	−0,39
ω3	FASTING BLOOD GLUCOSE	0,41	−0,20	−0,60	−0,20	0,22
	POSTPRANDIAL BLOOD GLUCOSE	−0,55	0,65	0,15	0,16	−0,18
ω3 + NDGA	FASTING BLOOD GLUCOSE	−0,43	−0,70	−0,32	−0,44	−0,36
	POSTPRANDIAL BLOOD GLUCOSE	0,22	−0,23	−0,27	−0,32	−0,20
WISTAR	FASTING BLOOD GLUCOSE	−0,70	0,84	0	0,11	0,18
	POSTPRANDIAL BLOOD GLUCOSE	−0,86	0.14	−0,29	0,16	0,12

*n* = 90 males rats (eSS control *n* = 10; ω-6 *n* = 21; ω-6 + NDGA *n* = 14; ω-3 *n* = 29; ω-3 + NDGA *n* = 16; Wistar rats non eSS *n* = 15) eSS control: Stillman Salgado rats without treatment; Wistar: non eSS rats without treatment. ω Groups: different PUFAs ω-3 and ω-6 treatments with or without NDGA (nordihydroguaiaretic acid)

GGT activity, which has an important role in SLGCI, was positively and significantly correlated to CRP. On the other hand, ω-6 treated group showed increased GGT and TG levels. CRP values presented a negative correlation, for both FBG and PBG in these groups. GGT and CRP levels decreased when NDGA was supplemented. Plasma TGs did not change with the addition of NDGA and in fact, a positive correlation was found both for FBG and PGB. In both ω-3 and ω-6 LCPUFAs treated groups without NDGA supplementation, GGT and CRP increased at the time of fasting but decreased at postprandial stage as recorded by correlation tests. Based on these results, it is suggested that supplementation of NDGA along with ω-3 could be beneficial to suppress insulin resistance, oxidative stress and inflammatory responses, at least in this experimental model of type 2 DM [48].

Previous works showed that both ω-3 and ω-6 LCPUFAs prevent alloxan-induced apoptosis of RIN (rat insulinoma) cells in vitro and alloxan-induced type 1 DM that is not mediated by both COX and LOX inhibitors indicating that prostaglandins and leukotrienes do not have any role in this cytoprotective action of fatty acids [49–52]. These results imply that a deficiency of ω-3 EPA and DHA and ω-6 AA may predispose to the development of DM. Type 1 and type 2 DM patients, as mentioned above, have decreased concentrations of AA, EPA and DHA and other unsaturated fatty acids in their plasma [53, 54] lending further support to the concept that unsaturated fatty acids may have a significant role in the pathobiology of DM. In a recent study, we noted that anti-inflammatory product of AA, lipoxin A4 (LXA4) concentrations are low in the plasma of patients with type 2 DM [55] suggesting that one mechanism by which LCPUFAs are able to prevent DM, or mitigate their complications, is by producing LXA4 and other similar anti-inflammatory products.

## Conclusions

In the spontaneous eSS rat type 2 DM model, as shown in the present study, ω-3 LCPUFAs are more effective than ω-6 in suppressing pro-inflammatory and oxidative stress markers seen in DM. These results are in agreement with the evidence that diets rich in ω-6 LCPUFAs such as red meat enhances circulating IL-6 levels and causes hyperglycemia and hyperlipidemia contributing to insulin resistance [56], though this is still debated. Hence, it is essential that a balance is maintained between ω-6/ω-3 LCPUFAs by consuming more amounts of ω-3 (mainly present in marine fish such as EPA and DHA) in the diet or by oral supplementation of fish oil capsules that may aid in suppressing inflammatory and oxidative stress indices and enhance insulin sensitivity and improve endothelial function [5, 43]. In this context, it is interesting to note that supplementation of both ω-3 and ω-6 PUFAs especially in combination with NDGA (while ω-6 PUFAs alone failed) inhibited hepatic IL-6 levels compared to eSS control (see Fig. 13) and these results are in line with the previous report that ω-3 PUFAs significantly reduces liver oxidative stress induced by high fat diet [57]. In addition, the observation that addition of NDGA has accentuated the beneficial actions of both ω-3 and ω-6 PUFAs in the presence of NDGA is rather interesting. Though we interpreted this beneficial action of NDGA in terms of its LOX inhibitory property, we are aware to the possibility that NDGA might be bringing about its useful actions by virtue of its antioxidant properties which are related to its ability to modulate Nrf2/ARE (nuclear factor erythroid 2-related factor 2/antioxidant response element) antioxidant pathway [58].

In a similar fashion, the beneficial actions of ω-3 and ω-6 PUFAs observed in the present study may also be due to the ability of these PUFAs to bypass the inhibitory action of high fat diet on enzymes desaturases [59] and upregulation of PPARs and inhibition of NF-kB by these unsaturated fatty acids [60]. Thus, the beneficial actions of various PUFAs are rather complex that need to be dissected in future studies

In the present study, we observed that NDGA supplementation along with ω-3 LCPUFAs is better suited to modify metabolic and inflammatory parameters that may be beneficial in restricting the progression of DM in eSS rats and its associated complications. The beneficial actions seen with the addition of NDGA along with ω-3 and possibly with ω-6 LCPUFAs could be related to the preferential formation of anti-inflammatory compounds from EPA and DHA and AA such as lipoxins, resolvins and protectins as proposed previously [5, 43]. However, these proposals need confirmation in future studies.

On the basis of the present results, we conclude that eSS rat type 2 DM model is useful to conduct studies as to the involvement of PUFAs and their metabolites in the pathophysiology of type 2 DM and their involvement of inflammatory process and oxidative stress events.

## Methods

### Experimental design

A total of 105 male rats were used, of which 15 were Wistar and 90 were eSS. After weaning, forty days old rats were randomly assigned to different groups as shown in Table 4. All animals were fed *ad libitum* with chow diet. Treatments were given once in a month for twelve months. All experimental animals received 0.40 ml (total volume) of isotonic saline solution (SS) added with PUFAs dissolved in 0.5% of ethanol and NDGA, as detailed in Fig. 4. Doses used were selected based on previous experiments and published literature [61]. All biochemical studies were performed at day 40 (before the start of PUFAs treatment) designed as breastfeeding weaning period, it was previously suggested that both obesity and type 2 DM may have their origins in the perinatal period, and at the end of 6 and 12 months of age [62, 63]. Wistar and a set of eSS rats received only saline solution formed the control groups. Blood samples were obtained from tails puncture of the animals for biochemical studies. After extraction, whole blood underwent centrifugation at 1500 RPM by 10 min and sodium citrate (70%) was used as anti-coagulant. Samples were kept at −80 °C freezer. This is supported by the results of the present study shown in Fig. 4, where it is seen that fasting plasma glucose levels estimated on day 40 (breastfeeding weaning period) are higher in control eSS rats compared to all other groups.

**Table 4:** Various experimental groups used in the study are shown

Groups	Wistar non eSS	eSS control	ω6 (6.25 mg/kg monthly)	ω-3 (6.25 mg/kg monthly)	ω6 + NDGA (6.25 mg/kg AA + 1.9 mg/kg NDGA monthly)	ω3 + NDGA (6.25 mg/kg DHA/EPA + 1.9 mg/kg NDGA monthly)
Total *n* = 105	*n* = 15	*n* = 10	*n* = 21	*n* = 29	*n* = 14	*n* = 16

*AA* Arachidonic acid, *EPA* Eiocsapentaneoic acid, *DHA* Docosahexaenoic acid

*n* = number of animals

Blood glucose was assayed in the venous blood with a glucometer (Accu-chek Performa®) monthly. Glycosylated Hemoglobin (A1C) was estimated by A1c Now ® (Bayer) meter. Serum triglyceride (TG) was assayed by enzymatic colorimetric method. Plasma ultra-sensitive CRP (hs-CRP), lipo- and -hydro peroxides, nitrites, and plasma gamma-glutamyl transpeptidase (GGT) were determined by colorimetric methods [64]. IL-6 levels were measured by ELISA (DO 450 and 570 nm) at 12 months of age in plasma and organs samples (as per tissue weight). At the end of 12 months, rats were euthanized by overdoses of isofluorane and tissue samples were obtained, homogenized, and processed for GLC, IL-6 determination and other assays.

### Oral glucose tolerance test (OGTT)

Animals were fasted for 8 h prior to this test. Fasting blood sample was obtained for glucose estimation and a second sample was obtained 2 h after the administration of oral glucose solution (1.75 g/Kg).

### Total plasma fatty acids profile determined by Gas chromatography

The lipids were extracted by Folch’s method and methylated with sodium methoxide. The separation, quantification and identification of fatty acid methyl esters (FAME) was performed using a capillary column (BPX 20 m longitude, 0.25 mm ID, 0.25 μm film, SUPELCO®, USA) in a Clarus 500® (Perkin-Elmer) gas chromatograph. The FAMEs were identified using a commercial standard (Nu-check®, USA). All values are expressed as % Area of total.

### Statistical analysis

The results are expressed as mean ± SE. Comparisons between multiple groups were performed by one-way ANOVA or Kruskall-Wallis test followed by Dunn’s post hoc test. The paired Student’s *t* test was used to analyze results of blood glucose test. Correlations between groups were determinate by Pearson Test, analysis of covariance and correlation test. Statistical significance was *P* < 0.05. All statistical tests were performed using INFOSTAT 3.1 and GRAPHPAD PRISM 5 software.

## Abbreviations

A1c: Glycosylated hemoglobin
COX: Cyclooxygenase
CRP: Plasmatic C reactive protein
DM: DM mellitus
eSS rats: Stillman Salgado Rats
FBG: Fasting blood glucose
FFA: Free fatty acid
GGT: Plasmatic Gamma Glutamyl Transpeptidase
GLC: Gas liquid chromatography
LGCI: Low-grade systemic chronic inflammation
LOX: Lipoxygenase
MUFAs: Mono unsaturated fat acid
NDGA: Nordihydroguaiaretic acid
PBG: Postprandial blood glucose
PUFAs: Polyunsaturated fatty acid
ROS: Reactive oxygen species
SFA: Saturated fatty acid
TAK1: Transforming growth factor β-activated kinase 1
TGs: Plasmatic triglycerides
